# Modulation of MicroRNA-194 and Cell Migration by HER2-Targeting Trastuzumab in Breast Cancer

**DOI:** 10.1371/journal.pone.0041170

**Published:** 2012-07-19

**Authors:** Xiao-Feng Le, Maria I. Almeida, Weiqun Mao, Riccardo Spizzo, Simona Rossi, Milena S. Nicoloso, Shu Zhang, Yun Wu, George A. Calin, Robert C. Bast

**Affiliations:** 1 Department of Experimental Therapeutics, the University of Texas MD Anderson Cancer Center, Houston, Texas, United States of America; 2 Life and Health Sciences Research Institute, School of Health Sciences, University of Minho, Braga, Portugal; 3 Department of Pathology, the University of Texas MD Anderson Cancer Center, Houston, Texas, United States of America; University of South Alabama, United States of America

## Abstract

Trastuzumab, a humanized monoclonal antibody directed against the extracellular domain of the HER2 oncoprotein, can effectively target HER2-positive breast cancer through several mechanisms. Although the effects of trastuzumab on cancer cell proliferation, angiogenesis and apoptosis have been investigated in depth, the effect of trastuzumab on microRNA (miRNA) has not been extensively studied. We have performed miRNA microarray profiling before and after trastuzumab treatment in SKBr3 and BT474 human breast cancer cells that overexpress HER2. We found that trastuzumab treatment of SKBr3 cells significantly decreased five miRNAs and increased three others, whereas treatment of BT474 cells significantly decreased two miRNAs and increased nine. The only change in miRNA expression observed in both cell lines following trastuzumab treatment was upregulation of miRNA-194 (miR-194) that was further validated *in vitro* and *in vivo*. Forced expression of miR-194 in breast cancer cells that overexpress HER2 produced no effect on apoptosis, modest inhibition of proliferation, significant inhibition of cell migration/invasion *in vitro* and significant inhibition of xenograft growth *in vivo*. Conversely, knockdown of miR-194 promoted cell migration. Increased miR-194 expression markedly reduced levels of the cytoskeletal protein talin2 and specifically inhibited luciferase reporter activity of a talin2 wild-type 3′-untranslated region, but not that of a mutant reporter, indicating that talin2 is a direct downstream target of miR-194. Trastuzumab treatment inhibited breast cancer cell migration and reduced talin2 expression *in vitro* and *in vivo*. Knockdown of talin2 inhibited cell migration/invasion. Knockdown of trastuzumab-induced miR-194 expression with a miR-194 inhibitor compromised trastuzumab-inhibited cell migration in HER2-overexpressing breast cancer cells. Consequently, trastuzumab treatment upregulates miR-194 expression and may exert its cell migration-inhibitory effect through miR-194-mediated downregulation of cytoskeleton protein talin2 in HER2-overexpressing human breast cancer cells.

## Introduction

HER2 is amplified and overexpressed in 25–30% of human breast cancers. Treatment with trastuzumab (Herceptin®), a humanized murine anti-HER2 monoclonal antibody [1], produced an objective response rate of 15% in extensively pretreated patients [2] and in 26% of previously untreated women with HER2-overexpressing metastatic breast cancer [3]. Trastuzumab in combination with chemotherapy was significantly more effective than chemotherapy alone in prolonging the time to disease progression, duration of response and overall survival [4], [5], [6], [7], resulting in its approval by the FDA in 1998. Trastuzumab provided one of the first deliberately targeted therapies for a human cancer and has now become the first-line treatment of choice for patients with primary or recurrent HER2-overexpressing breast cancer [8].

Trastuzumab-based combination treatment with chemotherapy are, however, effective in 60–70% of the HER2-overexpressing breast cancers [7], [9] and trastuzumab resistance often develops during the course of treatment [9], [10]. Improving response to trastuzumab and reducing trastuzumab resistance is a critical and urgent priority for improving the clinical management of a substantial subset of patients with breast cancer. While significant progress has been achieved, molecular mechanisms underlying the action of trastuzumab are still not fully elucidated. Treatment with trastuzumab inhibits proliferation of breast cancer cells by blocking the phosphatidylinositol-3-kinase (PI3K) signaling pathway, upregulating the cyclin dependent kinase inhibitor p27Kip1 and inducing G1 arrest of the cell cycle [11], [12], [13], [14], [15], [16], [17], [18], [19], [20]. We and others have also illustrated the ability of trastuzumab to interfere with DNA repair [14], [21] and tumor angiogenesis [22], [23], [24], [25], [26], [27]. Others have provided evidence that trastuzumab can cleave the extracellular domains of HER2 receptors [28], [29], mediate antibody-dependent cellular cytotoxicity [30], [31], [32], and can induce apoptosis [33], [34], [35].

Despite these many observations, the effects of trastuzumab on regulation of cellular processes by microRNA (miRNA) are still not investigated in depth. miRNAs are small non-coding RNAs of 19–25 nucleotides that can negatively regulate gene expression at post-transcriptional and transcriptional levels [36], [37]. The binding of miRNAs to complementary sites in the 3′- untranslated regions (UTRs) and other regions of protein-coding mRNA sequences cause either degradation of the mRNA or inhibition of translation [36], [37]. miRNAs have been demonstrated to regulate many normal physiological processes [36], [37], to participate in malignant transformation at many sites including breast cancer [38], [39], [40], and to mediate sensitivity to chemotherapy and radiotherapy [41], [42], [43]. In this report, we have measured the effect of trastuzumab on miRNAs and the role of miRNAs in trastuzumab-mediated regulation of human breast cancer cells that overexpress HER2. We have found that treatment with trastuzumab affected expression of several miRNAs. miR-194 was upregulated and downregulated talin2, inhibited cell migration and invasion, which may contribute to the anti-tumor activity of trastuzumab on HER2-overexpressing breast cancer cells.

## Materials and Methods

### Cell lines and cell culture

Human breast cancer cell lines BT474, SKBr3, MDA-MB-361 and MDA-MB-231 were originally purchased from the American Type Culture Collection (Manassas, VA) and stored, recovered and used at early passage from cryopreservation in liquid nitrogen. SKBr3, MDA-MB-361 and MDA-MB-231 cells were maintained in RPMI 1640 medium containing 10% fetal bovine serum (FBS), 2 mM L-glutamine, penicillin (100 units/mL), and streptomycin (100 µg/mL). BT474 cells were grown in complete medium containing DMEM supplemented with 10% FBS, 2 mM L-glutamine, 1 mM sodium pyruvate (Sigma, St. Louis, MO), 100 units/ml of penicillin, and 100 μg/ml streptomycin. All cell culture media were obtained from the Media Preparation Core Facility at the University of Texas MD Anderson Cancer Center. All cell lines were cultured in a humidified air supplemented with 5% CO_2_ at 37°C and tested monthly for mycoplasma with a MycoSensor PCR Assay Kit from Stratagene (Cat# 302109, La Jolla, CA). All cell lines were authenticated by the Cell Line Core Facility at the University of Texas MD Anderson Cancer Center with short tandem repeat DNA fingerprinting in April, 2011.

### Reagents

Trastuzumab (Herceptin®) manufactured by Genentech Inc (South San Francisco, CA) was purchased from Division of Pharmacy at the University of Texas MD Anderson Cancer Center. Human immunoglobin G (hIgG) served as control for trastuzumab and was purchased from Calbiochem (La Jolla, CA). Antibodies reactive with vinculin and total Rb were purchased from Cell Signaling Technology, Inc. (Beverly, MA). Antibody against talin2 was purchased from Santa Cruz Biotechnology, Inc. (Santa Cruz, CA). miR-194 precursor and its miRNA negative controls, miR-194 inhibitor and its negative inhibitor, and TaqMan assay for miR-194 were purchased from Applied Biosystems Incorporated (ABI, Foster City, CA). Small interfering RNAs (siRNAs) targeted to talin2 and talin1 were from Dharmacon (Lafayette, CO) or Ambion (Austin, TX). Epidermal growth factor (EGF) used in cell culture was ordered from Sigma (St. Louis, MO). Transfection reagents used were Lipofectamine 2000 from Invitrogen (Grand Island, NY) and DharmaFECT #4 from Dharmacon.

### Preparation of Total RNA

Total RNA was extracted with the TRIzol reagent (Invitrogen, Carlsbad, CA) according to the manufacturer's instructions. RNA purity was assessed by measuring absorption at 260 nm (A260) and at 280 nm (A280) (samples that had A260/A280 ratio of 1.9–2.1 were considered acceptable) and by ethidium bromide staining of 18S and 28S RNA on gels after electrophoresis. RNA concentrations were determined from the A260. Before performing the microRNA assay, each RNA sample was treated by DNase with a Turbo DNase kit (Ambion, Austin, TX).

### miRNA Microarray Profiling

miRNA profiling was performed in the non-coding RNA core facility at M.D, Anderson Cancer Center. Briefly, 5 µg of RNA from each tissue sample was labeled with biotin by reverse transcription using random octomers in duplicate. Hybridization was carried out on the second version of a miRNA-chip A-MEXP-1246 version (http://www.ebi.ac.uk/arrayexpress/arrays/A-MEXP-1246), which contained 559 human and 393 mouse miRNA probes, 962 human ultraconserved sequences in quadruplicate. Each oligo was printed in duplicate in two different slide locations. Hybridization signals were detected by biotin binding of a Streptavidin-Alexa647 conjugate (one-color signal) using a GenePix 4000B scanner (Axon Instruments). We quantified images using the GenePix Pro 6.0 (Axon Instruments). Raw data were analyzed in BRB-ArrayTools developed by R. Simon and A.P. Lam (version: 4.2.1, National Cancer Institute). Expression data were normalized by quantiles method of the Bioconductor package. Statistical comparisons were done using BRB class comparison *t*-test. A total of 16 samples from BT474 cells (8 from wild-type or trastuzumab-sensitive cells and another 8 from trastuzumab-resistant cells) and 8 samples from SKBr3 cells (4 from wild-type or trastuzumab-sensitive cells and another 4 from trastuzumab-resistant cells) were analyzed. Samples were discarded if more than 87.5% and 75% for BT474 and SKBr3 cell line, respectively, of missed values occurred (the miRNAs are retained when present in at least the smallest group in the dataset), missed values were replaced with 4.500001 (basic expression value, log2) after normalization. miRNAs significant at 0.05 level of the univariate test were reported. This microRNA microarray data regarding breast cancer cell lines treated with or without trastuzumab have been deposited into the ArrayExpress (http://www.ebi.ac.uk/miamexpress/).

### Quantitative Reverse Transcription–Polymerase Chain Reaction (QRT-PCR) Analysis

QRT-PCR was performed to assess mir-194 levels with the use of a Prism 7900HT Sequence Detection System (ABI, Foster City, CA) and TaqMan Real-Time PCR Assay (ABI). Total RNA was reversely transcribed into complementary DNA (cDNA) with specific stem-loop RT primer and a cDNA kit from ABI. Hsa-miR-194 was purchased from ABI (Assay ID 000493). Amplifications were carried out in triplicate on MicroAmp optical 96-well microliter plates (ABI). Thermal cycling conditions were as follows: 95°C for 5 minutes, followed by 40 cycles of 95°C for 10 seconds and 59°C for 40 seconds. U6 RNA was used as an internal control in all QRT-PCR assays for miRNA. The ΔΔC_T_ method was used to compare the relative expression levels between treatments. The final PCR results were expressed as the relative expression compared to individual control sample in each assay.

### RNA Blotting

miR-194 Northern blotting was performed as described previously [44]. Briefly, total RNA isolation was performed using the Tri-Reagent (Invitrogen). RNA samples of 25 µg each were run on 15% acrylamide denaturing (urea) criterion precast gels (Bio-Rad), and then transferred onto Hybond-*N*_membrane (Amersham Pharmacia Biotech). The hybridization with a DNA oligonucleotide probe complementary to the mature miR-194 sequence end-labeled with [γ-^32^P] ATP was performed at 42°C in 7% SDS/0.2 M Na_2_PO_4_ (pH 7.0) overnight. The membrane was washed twice at 42°C with 2× standard saline phosphate EDTA (SSPE, 0.18 M NaCl/10 mM phosphate, pH 7.4/1 mM EDTA)/0.1% SD S and twice with 0.5× SSPE/0.1% SDS. The blot was stripped by boiling in 0.1% SDS/0.1× SSC for 10 min and was reprobed U6 snRNA as a loading control.

### miRNA Transfection

BT474 or SKBr3 cells were seeded on 6-well culture plates and transfected with a negative control sequence or miR-194 using the DF4 reagent (Dharmacon). The mixture of miRNA (15 nM final concentration) and DF4 reagent (12.5 nM final concentration) were incubated for 20 min before being added to cells. Transfection was carried out for 72 hrs and cells were harvested for extraction of RNA or protein.

### Cell Proliferation Assays

A crystal violet cell proliferation assay was used to assess anchorage-dependent cell proliferation as described previously [15]. Briefly, BT474 stable cells that either express empty vector or miR-194 were seeded and cultured in triplicate in 96-well cell culture plates for different intervals. The cells were washed with phosphate-buffered saline, fixed in 1% glutaraldehyde, and stained with 0.5% crystal violet (Sigma Chemical Co.) dissolved in methanol. The dye that stained the cells on the plates was then eluted with Sorenson's buffer (0.9% sodium citrate, 0.02 N HCl, and 45% ethanol), and directly measured with the use of a Vmax microplater reader (Molecular Devices, Sunnyvale, CA) at a wavelength of 560 nm. Cell number was expressed as the optical density (OD) at 560 nm.

### BT474 Human Breast Cancer Xenografts in Nude Mice

The BT474 xenograft model was established in female nu/nu Balb/c mice as described previously [22] . Briefly, BT474 wild-type cells or stable transfectants expressing miR-194 were injected subcutaneously into two mammary fat pads of 4-week-old BALB/c athymic Nu/Nu mice (obtained from the in-house animal facility at the Department of Experimental Radiation Oncology, the University of Texas MD Anderson Cancer Center). Experiments with nu/nu mice were reviewed and approved by the Institutional Animal Care and Use Committee at the University of Texas MD Anderson Cancer Center. All mice were maintained under specific pathogen-free conditions and given sterile food and water. Once the tumors became palpable (at day 14 after injection), the mice were randomly divided into two treatment groups (6 mice per group): 1) intraperitoneal injection with control hIgG (1 mg/kg) twice a week for 3 weeks.; or 2) intraperitoneal injection with trastuzumab (1 mg/kg) twice a week for 3 weeks. At the end of treatment, all tumors were collected and weighed. A portion of the xenografts was used for RNA and protein isolation; another portion was fixed in formalin and embedded in paraffin; and third portion was frozen in liquid nitrogen. Experiments with nude mice were repeated twice with similar results.

### miR-194 Expression Construct

A miR-194 core fragment was amplified from BT474 genomic DNA by using the following primer set: CTAAGCTTAGTGGGCATGGGACACTCT (miR-194-2F) & CTGAATTCACCTGCCTCTCCTTCTTCGT (miR-194-2R) and subcloned into pEGPF-C1 vector digested with HindIII and EcoRI. The sequence of this miR-194 construct was verified by direct sequencing and QRT-PCR after transient expression.

### Generation of Trastuzumab-resistant Cells and Stable Clones that Express miR-194 in BT474 and SKBr3 cells

Trastuzumab-resistant SKBr3 and BT474 cells were generated as reported previously [45]. Two stable clones that express miR-194 and two control clones that express the backbone vector (pEGPF-C1) were established in BT474 cells using previously reported methods [46] .

### Generation of 3′ UTR reporter constructs of talin2

Prediction of miR-194 binding sites was performed using TargetScan software (http://www.targetscan.org/). A fragment of 3′-′UTR region of the talin2 contains the predicted binding site for miR-194 and was amplified by PCR using the primers: TCTAGA GGCTGCATGATCGTGATGT (forward) and TCTAGA TCATAAAGAGGTCCAGGAGCA (reverse), which contained Xba I restriction sites (underlined nucleotides). The PCR product was purified, digested and cloned into pGL3 vector (Promega, Madison, WI) via the Xba I site, which is located downstream of the firefly luciferase reporter gene. QuikChange® II XL Site-Directed Mutagenesis Kit (Agilent Technologies, Santa Clara, CA) was used to generate a deletion mutation in the miR-194 seed region according to the manufacturer's instructions. Following mutagenic primers: 5′-GGGTGCACGTTTCATGGACAACAAAGAAAAGTCAGT-3′ (deletion sense) and 5′-ACTGACTTTTCTTTGTTGTCCATGAAACGTGCACCC-3′ (deletion antisense) were utilized. Generated constructs were confirmed by direct sequencing using an ABI 3730xl DNA sequencer at the DNA Analysis Core Facility at the University of Texas MD Anderson Cancer Center.

### Dual Luciferase Reporter Assay

Luciferase activity assays were performed as previously reported [46] . Briefly, cells were seeded in 6-well plates, cotransfected with miR-194 precursor or its negative control and a wild-type or mutated talin2 3′-UTR reporter construct as described above. A *Renilla* luciferase vector (pRL-TK) served as an internal control and was included in all samples. After transfection for 16 hrs, cells were split into 12-well plates, harvested after 24 hrs and Firefly and Renilla luciferase activities were measured sequentially using the dual luciferase assay kit (Promega) and a luminometer. Results were expressed as relative luciferase activity after normalization with *Renilla* luciferase activity. Results represent three independent experiments and each performed in triplicate.

### Immunoblot Analysis

Total cell lysates were prepared and Western blotting was performed as described previously [15]. Briefly, cells were transfected with miR-194 precursors for 3 days, and then harvested for total lysate preparation. Total lysates were separated on 6% SDS-polyacrylamide gel and blotted onto nitrocellulose membrane. The membrane was incubated with horseradish peroxidase–conjugated secondary antibody (1∶2000; GE healthcare) and bound antibody was visualized with the use of a SuperSignal West Dura chemiluminescent kit (Thermo Fisher, Rockford, IL).

### Flow Cytometry

The percentage of the sub-G1 cell population (apoptotic cells) and the cell cycle distribution were determined based on relative DNA content with the use of flow cytometry as described previously [15].

### Cell Migration Assay

2×10^5^ of BT474 cells or 5×10^4^ SKBr3 cells in 0.5 ml of serum-free medium were introduced into the upper compartment of the BD BioCoat control inserts (Cat. # 354578, BD Discovery Labware, Bedford, MA) fitted with membranes of 8 micron porosity separating the upper and lower compartments. The lower compartment was filled with normal culture medium, medium supplemented with 10% FBS. After 16 hrs of incubation, cells were wiped off from the upper surface of each insert. The cells on the lower surface, which represented the cells that migrated through control insert membrance, were fixed and stained with Diff-Quick (Siemens, Deerfield, IL) and counted by microscopic examination in 10 representative fields. Cell migration was expressed as relative migration relative to the migration of each control group. Cell migration at each control group was arbitrarily set as 1. Each condition was assayed in triplicate and each experiment was repeated at least three times.

### Cell Invasion Assay

Invasion assays were performed using BD Biocoat Matrigel Invasion Chambers (Cat. # 354480, BD Discovery Labware, Bedford, MA) that contain an 8 micron pore size PET membrane with a thin layer of MATRIGEL Basement Membrane Matrix by following the manufacturer instructions. Briefly, control-treated or miR-194 (or trastuzumab)-treated BT474 or SKBr3 cells were introduced into the upper compartment, incubated for 24 hrs, fixed and stained after removing non-invading cells as described above for the Cell Migration Assay. Cell invasion is then calculated as the percent invasion through the matrigel matrix and membrane relative the migration through the control membrane. Cell invasion at each control group was arbitrarily set as 1. Cell invasion data was expressed as relative invasion relative to the invasion of control group.

### In Vitro Scratch Assay

BT474 (5×10^6^ cells/well) or SKBr3 (2×10^6^ cells/well) cells were seeded to 90% confluence in a 6-well plate for overnight culture. The following day a scratch was made through the center of each well using a 200-µL pipette tip, creating an open “scratch” or “wound” that was clear of cells. The dislodged cells were removed by three washes with complete culture media, and cells were incubated under standard conditions. Migration into the open area was documented at 72 hrs post-scratching.

### Vinculin Immunofluorescence (IF) Staining

SKBr3 cells were seeded at 1.5×10^5^ cells onto collagen (10 µg/ml) and poly-L-lysine (1 µg/ml) coated glass coverslips in 6-well dishes. Cells were grown for 16 hrs and then either cultured untreated or treated with control hIgG (10 µg/ml) or trastuzumab (10 µg/ml) for 16 hrs. Cells were fixed in ice-cold MeOH at −20°C for 5 min. Fixed cells were permeabilized and immunostained with rabbit anti-vinculin and followed by incubation with secondary antibody secondary antibody conjugated with Alexa Fluor 594 (Invitrogen). Coverslips were mounted on glass slides and examined using an Olympus FluoView FV1000 confocal microscope (Center Valley, PA).

### Ethics Statement

All animal experiments with nu/nu mice were reviewed and approved by the Institutional Animal Care and Use Committee at the University of Texas MD Anderson Cancer Center. Animals were euthanized when the mice became morbid or the tumor size is 1.5 cm in the largest diameter.

### Statistical Analysis

All experiments were repeated at least three times on different occasions. The results are presented as the mean ± SD for all values. A paired Student's *t* test was used to evaluate statistically significant differences in miR-194 levels between the treatment groups and the vehicle control group. *P*<0.05 was considered statistically significant. All statistical tests and corresponding *P* values were two-sided.

## Results

### Trastuzumab treatment upreguates miR-194 in HER2 overexpressing breast cancer cells

To determine the effects of trastuzumab treatment on miRNAs in HER2-overexpressing breast cancer cells, SKBr3 and BT474 cells were treated with trastuzumab or control hIgG. RNA was extracted and profiled on a miRNA chip (ArrayExpress) that contained 559 human miRNA probes and 962 human ultraconserved sequences. Raw data were analyzed using a GeneSpring GX 7.3. The filter on fold change was set at 1.2 since this threshold had been demonstrated to reflect a significant biological difference [47]. As shown in Table 1, trastuzumab treatment in SKBr3 cells increased 23 human miRNAs including miR-194 and miR-629, but decreased 33 human miRNAs in SKBr3 cells. In BT474 cells, trastuzumab treatment upregulated more miRNAs than it downregulated. As shown in Table 2, trastuzumab increased 40 human miRNAs including miR-194, decreased 18 human miRNAs in BT474 cells. To visualize the clusters of the differentially expressed miRNAs in response to trastuzumab treatment in the two cell lines, hierarchical clustering was performed using one minus correlation as a distance measure and average linkage method for defining the distance between the clusters (Fig. S1). When miRNA changes were compared in SKBr3 and BT474 breast cancer cells, miR-194 upregulation was the only change shared by both cell lines. Consequently, miR-194 was selected for further study.

**Table 1:** miRNAs affected by trastuzumab in SKBr3 cells.

Unique ID	Mean ratio of trastuzumab vs control hlgG	p-value (t-test)	Geom mean of intensities in SKBr3 cells treated with control hIgG for 20 hrs (BRC20)	Geom mean of intensities in SKBr3 cells treated with trastuzumab for 20 hrs (BRHCT20)
hsa-mir-548b-A	0.065146674	0.0002274	347.37	22.63
hsa-mir-551a-P	0.071772555	0.0185408	452.68	32.49
hsa-mir-624-A	0.072392834	0.0169737	312.6	22.63
hsa-mir-648-A	0.075340413	0.0148338	300.37	22.63
hsa-mir-544-A	0.076597617	1.10E-05	295.44	22.63
hsa-mir-26a-2-P	0.083364032	0.0067642	271.46	22.63
hsa-mir-508-A	0.086219377	0.0030508	262.47	22.63
hsa-mir-608-P	0.099895462	0.028476	325.24	32.49
hsa-mir-33-A	0.100908274	0.0283768	300.57	30.33
hsa-mir-519a1-5p/526c-A	0.103456158	2.57E-05	218.74	22.63
hsa-mir-433-P	0.103754986	0.0014047	218.11	22.63
hsa-mir-548a3-P	0.110535828	0.033025	204.73	22.63
hsa-mir-516-4-5p-A	0.116595394	0.0157548	194.09	22.63
hsa-mir-520g-P	0.132984662	0.01343	170.17	22.63
hsa-mir-526a-2-P	0.135202744	0.0372519	247.85	33.51
hsa-mir-192-P	0.174889247	0.0003376	397.28	69.48
hsa-mir-519b-3p/526c-A	0.180074799	0.0152054	125.67	22.63
hsa-mir-141-P	0.202469357	0.0036423	111.77	22.63
hsa-mir-153-2-A	0.215605945	0.0208085	104.96	22.63
hsa-mir-381-A	0.240514401	0.0338008	94.09	22.63
hsa-mir-613-P	0.264091493	0.0242617	85.69	22.63
hsa-mir-135b-A	0.277634646	0.0053856	81.51	22.63
hsa-mir-506-P	0.314426262	0.0432334	199.22	62.64
hsa-mir-616-P	0.362195903	0.0017904	62.48	22.63
hsa-mir-651-A	0.381449112	0.0349512	212.82	81.18
hsa-mir-496-P	0.410558781	0.0179086	55.12	22.63
hsa-mir-652-A	0.45070703	0.045327	50.21	22.63
hsa-mir-624-P	0.477062244	0.0097793	139.29	66.45
hsa-mir-200a*-5p-A	0.509914376	0.0301038	44.38	22.63
hsa-mir-135a-1-A	0.558314088	0.0331035	103.92	58.02
hsa-mir-196b-P	0.56347169	0.0475296	2699.55	1521.12
hsa-mir-214-A	0.697566648	0.0485026	1745.74	1217.77
hsa-mir-29a-A	0.713695323	0.0199925	1379.23	984.35
hsa-mir-629-A	1.215502868	0.0165149	1799.28	2187.03
hsa-mir-194-2-A	1.222424312	0.0168992	3056.95	3736.89
hsa-mir-27b-A	1.466522781	0.0174398	838.63	1229.87
hsa-mir-572-P	1.710230649	0.0482883	706.7	1208.62
hsa-mir-518a2-5p/mir527-A	2.068051259	0.0064793	22.63	46.8
hsa-mir-569-P	2.840919134	0.0007198	22.63	64.29
hsa-mir-17-3p-A	2.933274414	0.0304101	22.63	66.38
hsa-mir-431-A	3.683163942	0.0495408	22.63	83.35
hsa-mir-30d-P	3.899322034	0.0487109	29.5	115.03
hsa-mir-96-P	4.094521778	0.022245	44.54	182.37
hsa-mir-557-P	4.207247017	0.0134777	22.63	95.21
hsa-mir-105-2-A	4.314184711	<1e-07	22.63	97.63
hsa-mir-489-P	4.814453843	0.0087891	54.38	261.81
hsa-mir-505-P	5.205695142	0.0310113	29.85	155.39
hsa-mir-583-A	5.604502889	0.0038414	50.19	281.29
hsa-mir-640-P	6.07512152	6.07E-05	22.63	137.48
hsa-mir-548d1-A	8.575879397	0.0167225	29.85	255.99
hsa-mir-519a1-3p-A	9.625276182	4.25E-05	22.63	217.82
hsa-mir-605-A	11.04639859	0.0011691	22.63	249.98
hsa-mir-340-P	11.06934673	0.0302754	29.85	330.42
hsa-mir-198-A	11.44410075	0.0041912	22.63	258.98
hsa-mir-19b2-A	12.41228458	0.0020803	22.63	280.89
hsa-mir-518e-3p-A	13.12019443	0.002743	22.63	296.91

**Table 2:** miRNAs affected by trastuzumab in BT474 cells.

Unique ID	Mean ratio of trastuzumab vs control hlgG	p-value (t-test)	Geom mean of intensities in BT474 cells treated with control hIgG for 48 hrs (BTC48)	Geom mean of intensities in BT474 cells treated with trastuzumab for 48 hrs (BTHCT48)
hsa-mir-211-P	0.092615377	0.034884	382.01	35.38
hsa-mir-514-2&3-A	0.10147527	0.000624	223.01	22.63
hsa-mir-518e-5p/526c-A	0.116523351	0.019672	194.21	22.63
hsa-mir-452*-3p-A	0.116631449	0.034182	194.03	22.63
hsa-mir-518a2-5p/mir527-A	0.144231995	0.035946	156.9	22.63
hsa-mir-524*-5p-A	0.162257116	0.00944	139.47	22.63
hsa-mir-518b-A	0.173503028	0.012805	130.43	22.63
hsa-mir-105-2-P	0.209385572	0.012188	458.15	95.93
hsa-mir-520h-P	0.227391479	0.002751	99.52	22.63
hsa-mir-519e*-5p-A	0.242584266	0.002037	331.39	80.39
hsa-mir-646-A	0.242915414	0.023069	93.16	22.63
hsa-let-7g-P	0.257715522	5.90E-06	87.81	22.63
hsa-mir-323-A	0.291360886	0.002132	77.67	22.63
hsa-mir-517b-3p-A	0.291698891	0.009441	77.58	22.63
hsa-mir-432-5p-A	0.292150788	0.010767	77.46	22.63
hsa-mir-515-1-5p-A	0.305356902	7.16E-05	74.11	22.63
hsa-mir-548a1-A	0.500150716	0.017816	663.5	331.85
hsa-mir-671-A	0.780467508	0.030883	1862.64	1453.73
hsa-mir-758-P	1.203505577	0.010282	790.74	951.66
hsa-mir-485-3p-A	1.226628339	0.007659	1019.29	1250.29
hsa-mir-102-A	1.238857151	0.007481	1353.11	1676.31
hsa-mir-202-3p-A	1.29220961	0.03779	964.65	1246.53
hsa-mir-629-A	1.363440315	0.017991	2721.96	3711.23
hsa-mir-663-P	1.371448683	0.003362	1647.98	2260.12
hsa-mir-560-P	1.446595051	0.041021	831.29	1202.54
hsa-mir-296-A	1.45630605	0.010238	545.27	794.08
hsa-mir-324-5p-A	1.462267846	0.032113	1455.39	2128.17
hsa-mir-219-1-P	1.517478105	0.000529	17165.19	26047.8
hsa-mir-206-P	1.594267196	0.000415	577.03	919.94
hsa-mir-498-A	1.630858004	0.019047	783.33	1277.5
hsa-mir-19b2-A	1.699504866	0.038757	351.42	597.24
hsa-mir-194-2-A	1.828481053	0.00689	3749.44	6855.78
hsa-mir-219-2-P	1.868439905	0.008312	1298.19	2425.59
hsa-mir-429-P	1.895429772	0.031979	1099.07	2083.21
hsa-mir-564-P	2.086289374	0.018767	875.89	1827.36
hsa-mir-521-2-P	2.461776403	0.010133	22.63	55.71
hsa-mir-126-5p-A	2.653743792	0.002951	1067.1	2831.81
hsa-mir-199a*-3p-A	2.856385329	0.048539	22.63	64.64
hsa-mir-101-1/2-P	3.303137428	0.000142	22.63	74.75
hsa-mir-638-P	3.462660186	0.04302	22.63	78.36
hsa-mir-548a3-P	3.467962881	0.000185	22.63	78.48
hsa-mir-571-A	3.785682722	0.000292	22.63	85.67
hsa-mir-643-P	3.855501547	1.14E-05	22.63	87.25
hsa-mir-662-P	4.589482987	0.009066	22.63	103.86
hsa-mir-559-P	4.655766681	7.55E-05	22.63	105.36
hsa-mir-584-A	5.155103844	0.030886	22.63	116.66
hsa-mir-585-P	6.66195316	0.018554	22.63	150.76
hsa-mir-659-A	6.761655012	0.015715	85.8	580.15
hsa-mir-587-A	7.121078215	0.019825	22.63	161.15
hsa-mir-591-A	8.88054883	0.048778	37.17	330.09
hsa-mir-18b-P	8.956212523	0.023338	71.71	642.25
hsa-mir-152-A	13.43314501	0.036953	37.17	499.31
hsa-mir-642-P	17.71718957	0.006654	22.63	400.94
hsa-mir-21-P	17.86831639	0.004662	22.63	404.36
hsa-mir-567-P	22.46221829	0.007096	22.63	508.32
hsa-mir-337-A	24.40212108	0.008015	22.63	552.22
hsa-mir-652-A	27.14891737	2.90E-05	22.63	614.38
hsa-mir-136-A	32.47459125	0.005502	22.63	734.9

### Trastuzumab upreguates miR-194 in cell culture and in xenografts

**Figure 1:**
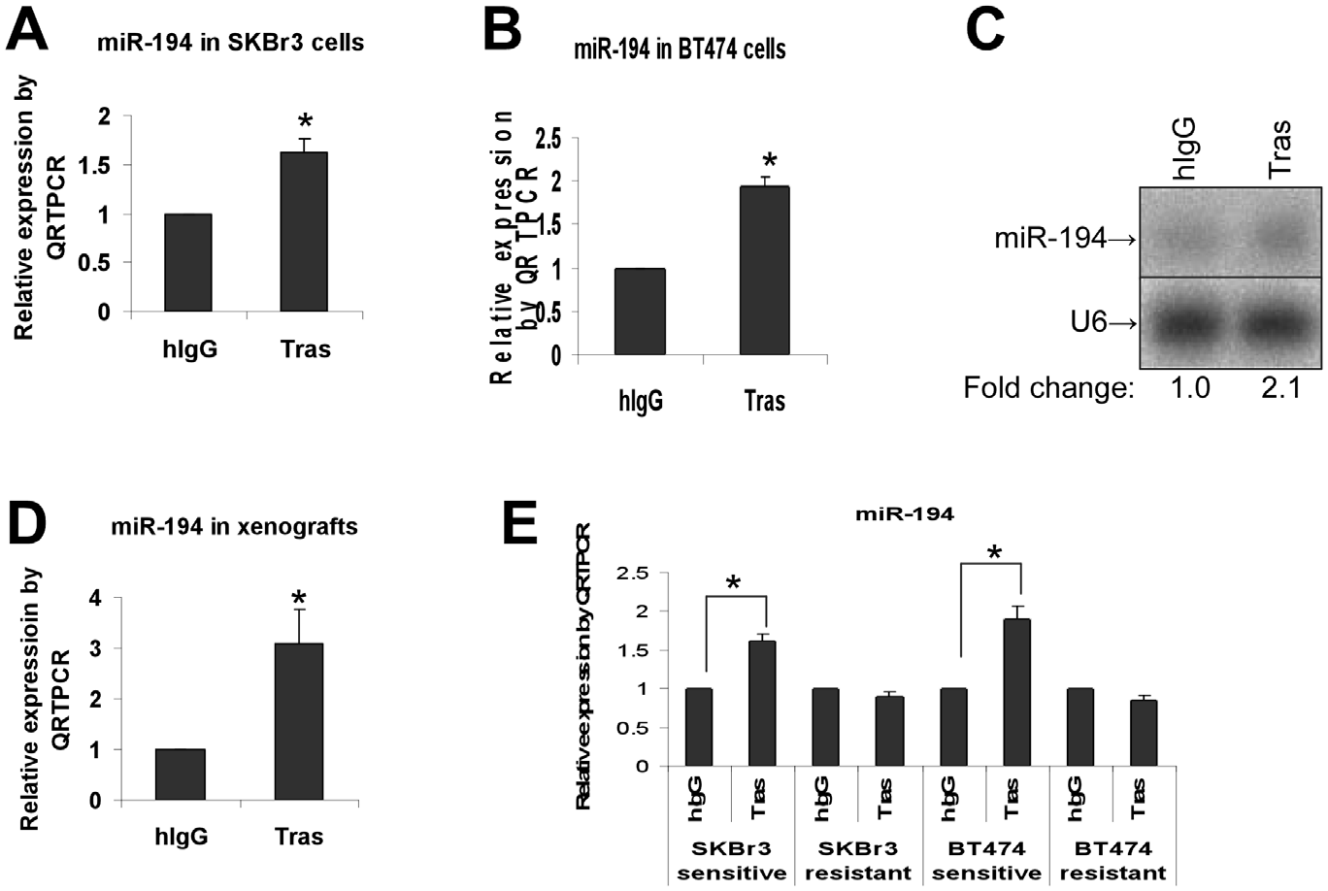
Trastuzumab upregulates miR-194 expression in vitro and in vivo. HER2-overexpressing breast cancer cell lines BT474 (**A**) and SKBr3 (**B**) were treated with trastuzumab (Tras) or control hIgG (10 µg/ml) for 48 hrs. Total RNA was prepared and analyzed by QRT-PCR to measure miR-194. * p<0.05 compared to hIgG control. (**C**) Northern blot analysis of miR-194 expression. BT474 cells were treated with Tras or control hIgG (10 µg/ml) for 48 hrs. Total RNA was prepared and analyzed by Northern blotting to detect miR-194. U6 non-coding small nuclear RNA (snRNA) served as a loading control. (**D**) QRT-PCR quantitation of miR-194 expression in vivo. BT474 xenografts in miR-194 levels were measured by QRT-PCR. * p<0.05 compared to hIgG control. (**E**) QRT-PCR quantitation of miR-194 expression in trastuzumab sensitive or resistant cell lines. Parental SKBr3 and BT474 (trastuzumab-sensitive), and their derived resistant cells were treated with Tras or control hIgG for 48 hrs. Total RNA was prepared and analyzed by QRT-PCR to detect miR-194. * p<0.05 compared to hIgG control.

To confirm that miR-194 was upregulated by trastuzumab (Tables 1 & 2), total RNA was extracted from trastuzumab-treated SKBr3 and BT474 cells in culture and subjected to quantitative reverse-transcription PCR (QRT-PCR) and Northern blot analyses. QRT-PCR analysis confirmed that trastuzumab treatment increased miR-194 by 1.64 fold in SKBr3 cells (Fig. 1A) and by 1.95 fold in BT474 cells (Fig. 1A). Northern blotting further confirmed that trastuzumab treatment increased miR-194 level by 2.1 fold in BT474 cells (Fig. 1C). An assay with a miR-194-specific sensor reporter also indicated that trastuzumab increased miR-194 expression (data not shown). Consistent with above results in cell culture, QRT-PCR analysis of BT474 xenograft tumor samples also showed that treatment with trastuzumab induced miR-194 expression by 3.1 fold compared to control hIgG (Fig. 1D). Collectively, above results confirm that trastuzumab induces miR-194 in cell culture and in vivo.

### Trastuzumab specifically induces miR-194 expression in trastuzumab-sensitive breast cancer cells

As shown in Figure 1E, miR-194 expression increased only in trastuzumab-sensitive SKBr3 and BT474 parental cells but not in trastuzumab-resistant cells. Additionally, miR-194 was not induced by trastuzumab treatment in either the MDA-MB-231 breast cancer cell line that expresses low levels of HER2 and is insensitive to trastuzumab or the KPL4 breast cancer cell line that expresses high levels of HER2 and is insensitive to trastuzumab (Fig. S2). Thus, miR-194 is specifically induced by trastuzumab treatment and may associate with trastuzumab response.

### Increased expression of miR-194 significantly inhibits migration and invasion of breast cancer cells that overexpress HER2

**Figure 2:**
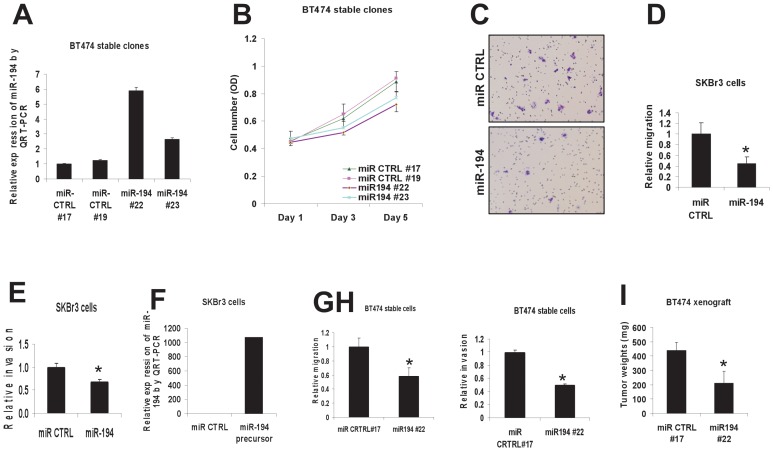
Increased miR-194 expression inhibits breast cancer cell migration and invasion. (**A**) miR-194 expression in the stable clones of BT474 cells. BT474 cells were stably transfected with an empty pEGFP-C1 vector or pEGFP-miR-194 vector under the selection of G418. Two control clones #17 and #19 that contain empty vector and two miR-194-expressing clones #22 and #23 were established and subjected for QRT-PCR analysis. Hsa-miR-194 was purchased from ABI (Assay ID 000493). (**B**) Cell viability assay of BT474 stable cells that express miR-194 or its control vector. BT474 cells were stably transfected with empty pEGFP-C1 vector or pEGFP-miR194 construct under the selection of G418. Two control clones #17 and #19 that contain empty vector and two miR-194-expressing clones #22 and #23 were chosen to measure viability of crystal violet-stained cells on day 1, day 3 and day 5. (**C**) Effect of miR-194 precursor on cell migration in SKBr3 cells. SKBr3 cells were transiently transfected with a miR-194 precursor or a control miRNA (miR CTRL) for 48 hrs and motility was measured overnight in a Transwell assay. (**D**) Quantitation of the SKBr3 cell migration as shown in (C). * p<0.05 compared to miR control. (**E**) Effect of miR-194 precursor on cell invasion in SKBr3 cells. SKBr3 cells were transiently transfected with a miR-194 precursor or a control miRNA (miR CTRL) for 48 hrs and invasion measured overnight. * p<0.05 compared to miR control. (**F**) miR-194 expression in transiently transfected SKBr3 cells. SKBr3 cells were transiently transfected with a miR-194 precursor or a control miRNA (miR CTRL) for 48 hrs. Total RNA was extracted and subjected to QRT-PCR analysis for miR-194 expression. Hsa-miR-194 was purchased from ABI (Assay ID 000493). (**G**) Assay of cell migration in BT474 stable cells that express miR-194 or a control vector. The control clone #17 and the miR-194-expressing clone #22 were chosen to study migration. * p<0.05 compared to #17 control. (**H**) Cell invasion assay in BT474 stable cells that express miR-194 or its control vector. The control clone #17 and the miR-194-expressing clone #22 were chosen to study invasion. * p<0.05 compared to #17 control. (**I**) BT474 xenograft tumor growth in vivo. BT474 xenografts in nude mice were established with the control clone #17 and the miR-194-expressing clone #22 as described in Methods. Tumors were collected and weighed after 4 weeks. * p<0.05 compared to #17 control.

We next explored the biological function of miR-194 in breast cancer. Two approaches were used to study the function of miR-194 in breast cancer cells: transient and stable expression of miR-194 in BT474 or SKBr3 breast cancer cells. The effects of transient and stable expression of miR-194 on cell viability, cell cycle, apoptosis and cell migration/invasion were evaluated. Stable expression of miR-194 in subclones of BT474 cells was confirmed with QRT-PCR and shown in Figure 2A. As shown in Figure 2B, stable overexpression of miR-194 in two BT474 stable clones (#22 and #23) produced moderate inhibition of cell growth as measured with a crystal violet viability assay. No significant change in cell cycle distribution or apoptosis was observed in subclones of BT474 with stable overexpression of miR194 (data not shown). Transient overexpression of miR-194 significantly decreased the ability of SKBr3 cells to migrate (Fig. 2C). Compared with the negative control miRNA, miR-194 expression reduced SKBr3 cell migration by 56% (Fig. 2D). Transient overexpression of miR-194 also decreased the ability of SKBr3 cells to detach from and invade through the matrigel matrix as illustrated in the cell invasion assay (Fig. 2E). Validation of miR-194 transient expression was performed with QRT-PCR and shown in Figure 2F. Additionally, stable overexpression of miR-194 inhibited the ability of BT474 cells to migrate by 40% and to invade by 55% (Fig. 2G & 2H). The effect of miR-194 on HER2-overexpressing breast cancer cells was further evaluated in a BT474 xenograft model. As shown in Figure 2I, miR-194 expressing BT474 tumors grew significantly less rapidly than did tumors containing breast cancer cells with an empty vector. Taken together, these data indicate that miR-194 can significantly inhibit cell migration/invasion in vitro, and tumor growth in vivo in breast cancer cells that overexpress HER2.

### miR-194 directly targets the talin2 gene and downregulates talin2 protein

**Figure 3:**
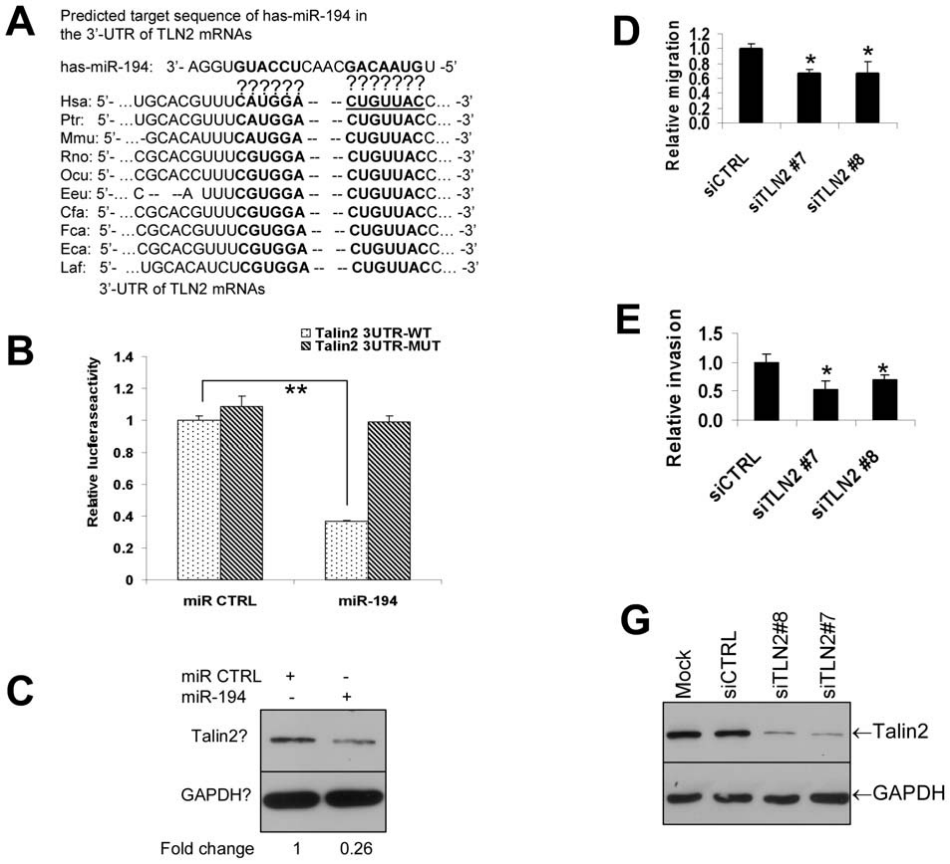
miR-194 targets the talin2 gene and downregulates talin2 protein levels. (**A**) Alignment of miR-194 with *talin2* (*TLN2*) 3′-UTRs. Complementary sequences of miR-194 and mammalian *talin2* 3′-UTRs are marked in Bold. The seed sequences of miR-194 are underlined. Has, human; Ptr, pan troglotydes; Mmu, mus musculus; Rno, rat; Ocu, rabbit; Eeu, hedgehog; Cfa, dog; Fca, cat; Eca, horse; Laf, elephant. The underlined seed nucleotides were deleted in the *talin2* 3′-UTR mutant reporter construct described in (B). (**B**) Effect of miR-194 expression on the luciferase activities of wild-type and mutated talin2 3′-UTR reporters. MDA-MB-361 cells were transiently transfected with a miR Control or miR-194 precursor for 36 hrs. Luciferase activity was determined using a dual luciferase assay. ** *p*<0.01. (**C**) Effect of miR-194 expression on talin2 protein levels in SKBr3 cells. SKBr3 cells were transiently transfected with a miR-194 precursor or a control miRNA (miR CTRL) for 48 hrs. Total protein was prepared and subjected to Western blotting. (**D**) Effect of talin2 downregulation on cell migration in SKBr3 cells. SKBr3 cells were transiently transfected with two siTalin2 (siTLN2 #7 and #8) or its control siRNA (siCTRL) for 48 hrs and then motility was measured overnight in a Transwell assay. * p<0.05 compared to siCTRL. (**E**) Effect of talin2 downregulation on cell invasion. SKBr3 cells were transiently transfected with two siTalin2 (siTLN2 #7 and #8) or its control siRNA (siCTRL) for 48 hrs and then invasion assay was performed. * p<0.05 compared to siCTRL. (**F**) Validation of talin2 siRNA efficacy. SKBr3 cells were transiently transfected with two siTalin2 (siTLN2 #7 and #8) or its control siRNA (siCTRL) or the transfection reagent only (mock) for 48 hrs and total protein was prepared. Western blotting was performed with a talin2 antibody.

We next asked whether miR-194 targeted the expression of proteins which regulate migration and invasion of breast cancer cells (Fig. 2). Based on the UCSC genome database at http://genome.ucsc.edu/, miR-194-2 maps on human chromosome 11 within an intron of an unknown human gene (AB429224, also called Homo sapiens cDNA FLJ35483). Using the TargetScan and miRanda programs, we identified talin2 that encodes a cytoskeletal protein as one of many genes targeted by miR-194. The putative miR-194 target site in the 3′-UTR of talin2 gene shows a close match to nucleotides 2 to 18 of miR-194 (Fig. 3A). The miR-194 target site is also highly conserved among multiple species including humans, chimpanzees, mice, rats, rabbits, hedgehogs, dogs, cats, horses and elephants (Fig. 3A). To determine whether miR-194 acts directly on talin2 3′-UTR, we conducted luciferase reporter assays, cotransfecting miR-194 and luciferase reporter constructs containing wild type (underlined letter in Figure 3A) or mutant (deletion of underlined letter in Figure 3A) talin2 3′-UTR. Luciferase activity was dramatically decreased by approximately 60% in the presence of miR-194 when compared with its negative miRNA control (Fig. 3B). In contrast, miR-194 did not alter activity of the mutant talin2 luciferase reporter that contained the deletion of miR-194 binding site (Fig. 3B), indicating miR-194 specifically act on wild-type talin2 3′-UTR. In agreement with the luciferase reporter results, transient overexpression of miR-194 significantly decreased talin2 protein expression (Fig. 3C). These data indicate that talin2 is a direct target of miR-194 in breast cancer cells.

### Depletion of talin2 inhibits cell migration and invasion in breast cancer cells that overexpress HER2

We next asked whether talin2 regulates cell migration and invasion. Both talin1 and talin2 encode high-molecular-weight cytoskeletal proteins that concentrate in focal adhesions and link integrins to vinculin and actin [48], [49]. To study the role of talin2 in cell migration, SKBr3 cells were transiently transfected with two siRNAs targeting talin2 or a negative control siRNA, and cell migration and invasion were assayed as described in Material and Methods. As shown in Figure 3D, knockdown of talin2 significantly inhibited migration of SKBr3 cells compared to the control siRNA. Talin2 silencing decreased cell invasion capacity as well (Fig. 3E). Two siRNAs used to downregulate talin2 was confirmed with Western blotting and shown in Figure 3F. Above data indicate that talin2 silencing decreases cell migration and invasion of SKBr3 cells.

### Trastuzumab treatment inhibits cell migration and disrupts the normal vinculin staining pattern in breast cancer cells that overexpress HER2

**Figure 4:**
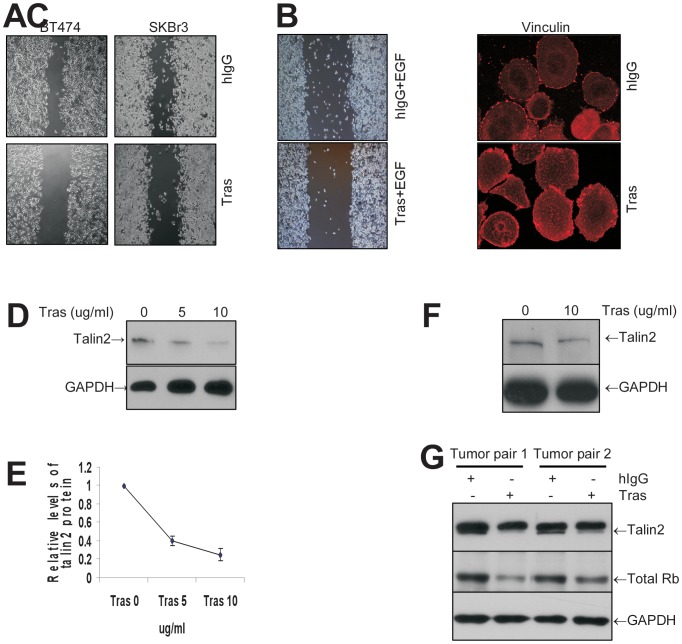
Trastuzumab treatment downregulates talin2 protein expression and inhibits breast cancer cell migration. (**A**) Effect of trastuzumab (Tras) on cell migration in a scratch assay. BT474 and SKBr3 cells were seeded in 6-well plates. After cells had grown to confluence, a scratch was made in the monolayer. Cells were treated with trastuzumab (Tras) or control hIgG (10 µg/ml) for 72 hrs. Images were recorded at 72 hrs at 40× enlargement. (**B**) Effect of trastuzumab on cell migration over a shorter interval (16 h) in a scratch assay. SKBr3 cells were seeded in 6-well culture plates and cultured overnight to achieve a cell density of full confluence. A scratch was made in the monolayer. Cells were then treated with trastuzumab (Tras) or control hIgG (10 µg/ml) plus epidermal growth factor (EGF, 20 ng/ml, sigma) for 16 hrs. Images were recorded at the end of 16 hrs of EGF stimulation at 40× enlargement. (**C**) Effect of trastuzumab (Tras) on cytoskeletal vinculin distribution after IF staining. SKBr3 cells were treated with trastuzumab (Tras) or control hIgG (10 µg/ml) for 48 hrs, and then subjected to IF staining as described in Methods. (**D**) Effect of trastuzumab on talin2 protein in BT474 cells. BT474 cells were treated in vitro with different concentrations of trastuzumab for 48 hrs. Total protein was prepared and subjected to Western blotting. (**E**) Quantitation of talin2 expression. The talin2 bands on immunoblots from three different experiments including the one shown in (**D**) were digitized, normalized to the levels of GAPDH, and expressed as mean levels (error bars correspond stand deviation). The talin2 expression at trastuzumab 0 concentration was set at 1. (**F**) Effect of trastuzumab on talin2 protein in SKBr3 cells. SKBr3 cells were treated with trastuzumab (Tras) or control hIgG (10 µg/ml) for 48 hrs. Total protein was prepared and subjected to Western blotting. (**G**) Effect of trastuzumab on talin2 protein in BT474 xenografts. BT474 xenografts in nude mice were treated with trastuzumab (Tras) or hIgG 1 mg/kg intraperitoneally twice a week and for 3 weeks. Total cell lysates were prepared and subjected to Western blotting.

While the effects of trastuzumab on cancer cell proliferation, angiogenesis, and apoptosis have been investigated in depth, the effect of trastuzumab on cell migration has received less attention. Both BT474 and SKBr3 cells respond to trastuzumab treatment, but are not actively motile. Long intervals are required to measure cell migration assay or to observe healing of scratches in cancer cell monolayers. Treatment of BT474 and SKBr3 with trastuzumab for 3 days significantly inhibited the ability of both cell lines to heal scratch assays (Fig. 4A). To study the effect of trastuzumab on cell migration over a shorter interval (16 h) and to minimize the effect of cell proliferation on scratch assays, we have stimulated SKBr3 cell migration with epidermal growth factor (EGF) and found that motility of trastuzumab-treated cells was significantly slower than that of hIgG-treated controls (Fig. 4B). Stimulation of SKBr3 cells with EGF for 16 hrs had no effect on miR-194 expression (data not shown). Similar results were observed in transwell cell migration assays (see below).

### Treatment with trastuzumab disrupts cytosekeltal organization

SKBr3 cells were treated with trastuzumab and stained by immunofluorescence for expression of vinculin, a cytoskeletal protein associated closely with talin2 and exhibiting a similar subcellular distribution [49]. In the absence of a specific antibody for talin2 for immunofluorescence studies, we have used the behavior of vinculin as a surrogate for talin2. As shown in Figure 4C, trastuzumab treatment for 16 hrs altered the staining pattern of vinculin. Instead of well ordered, discrete and focal staining of vinculin on the cell periphery, trastuzumab treatment produced disordered and clumpy staining both in the cytoplasm and at the cell's periphery. These observations suggest that trastuzumab can inhibit migration and disrupt the normal distribution of cytoskeletal proteins in breast cancer cells that overexpress HER2.

### Trastuzumab treatment downregulates talin2 protein expression in vitro and in vivo in breast cancer cells that overexpress HER2

From the data presented above, trastuzumab treatment increases miR-194 (Tables 1 & 2 and Fig. 1) and miR-194 negatively regulates cell migration (Fig. 2) and talin2 expression (Fig. 3). To determine whether trastuzumab downregulates talin2 expression, BT474, SKBr3 cells and BT474 xenograft tumors were treated with trastuzumab or control hIgG and total protein was collected, Western blots prepared and stained with anti-talin2. As shown in Figure 4D, trastuzumab treatment decreased talin2 protein expression in a dose-dependent manner in BT474 cells. Quantitation of talin2 protein levels after trastuzumab treatment in three different experiments confirmed the dose-dependent effect (Fig. 4E). Trastuzumab treatment also decreased talin2 protein in SKBr3 cells (Fig. 4F). A similar downregulation of talin2 was observed in BT474 xenografts growing in nude mice. Trastuzumab inhibited talin2 protein expression (the lower band) in two different tumors (Fig. 4G). Trastuzumab treatment also produced a decrease in total Rb protein in BT474 xenografts, consistent with activity of the antibody in vivo [15].

### Knockdown of miR-194 promotes cancer cell migration and reverses trastuzumab inhibition of cell migration

**Figure 5:**
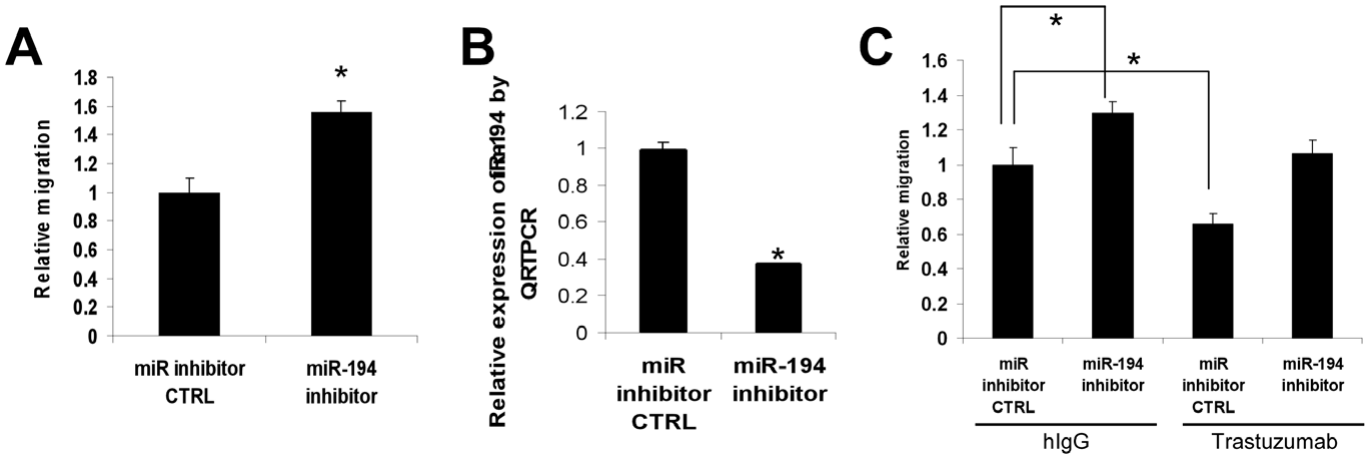
miR-194 inhibitor stimulates cell migration and blocks trastuzumab-inhibited cell migration. (**A**) Effect of miR-194 inhibitor on cell migration in SKBr3 cells. SKBr3 cells were transiently transfected with a miR-194 inhibitor or its negative control (miR inhibitor CTRL) for 48 hrs before measurement of migration overnight. * p<0.05 compared to the negative control. (**B**) Validation of efficacy of miR-194 inhibition. SKBr3 cells were transiently transfected with a miR-194 inhibitor or its negative control (miR inhibitor CTRL) for 48 hrs. Total RNA was prepared and miR-194 measured by QRT-PCR. * p<0.05 compared to miR inhibitor control. (**C**) Effect of miR-194 inhibitor on trastuzumab-inhibited cell migration. SKBr3 cells were transiently transfected with a miR-194 inhibitor or its negative control (miR inhibitor CTRL) for 16 hrs, treated with trastuzumab (Tras) or control hIgG (10 µg/ml) for 36 hrs, and motility was then measured for overnight in Transwell assays. * p<0.05.

To determine whether a decrease in miR-194 would increase motility, SKBr3 cells were transiently transfected with a specific miR-194 inhibitor or antagomir. As shown in Figure 5A, transfection of SKBr3 cells with a miR-194 antagomir increased cancer cell migration by more than 55%. The ability of the miR-194 antagomir to silence miR-194 was confirmed by QRT-PCR as shown in Figure 5B. Similar results were observed in BT474 cells, where inhibition of miR-194 by a miR-194 antagomir stimulated BT474 cell migration (Fig. 5C). Consistent with the results of *in vitro* scratch assay, trastuzumab treatment decreased the BT474 cell migration (Fig. 5C). However, trastuzumab treatment was no longer able to inhibit cell migration in the presence of miR-194 antagomir or inhibitor (Fig. 5C), indicating miR-194 induction was required for trastuzumab to slow cell migration in breast cancer cells.

## Discussion

In this study, we have found for the first time that miR-194 is induced in HER2-overexpressing breast cancer cells by trastuzumab treatment. Forced expression of miR-194 inhibits cell migration and decreases levels of talin2, a cytoskeletal protein. Treatment with trastuzumab also inhibits breast cancer cell migration and decreases talin2 expression. Depletion of miR-194 stimulates breast cancer cell migration and abolishes trastuzumab-inhibited cell migration.

The regulatory role of miR-194 was first studied in normal and malignant cells of the gastrointestinal tract. High levels of miR-194 are expressed in the intestines and liver [50], [51]. Hepatocyte nuclear factor (HNF) can induce miR-194 expression during intestinal epithelial cell differentiation [52], [53]. miR-194 suppresses invasion and migration of liver mesenchymal-like cancer cells [54]. miR-194 expression is elevated in normal colon tissues and low in colon cancers [55]. Low miR-194 expression has been associated with large tumor size and advanced stage in gastric cancer [56]. Activation of tumor suppressor p53 can induce miR-194 and its clustered miR-192 and miR-215 expression [55], [57]. miR-192 and miR-215 can induce p21Cip1 and cell cycle arrest in colon cancer cells [55]. In endometrial cancer cells, miR-194 has been reported to inhibit self-renewal factor BMI-1, reduce cell invasion and inhibit epithelial-mesenchymal transition (EMT) [58]. While inhibition of cell migration by miR-194 has been demonstrated in other systems, this is the first report to show that miR-194 can inhibit breast cancer cell migration (Fig. 2) and that miR-194 and migration can be regulated by trastuzumab.

Our data suggest that miR-194-induced inhibition of motility is mediated by downregulation of cytoskeletal proteins. We have demonstrated for the first time that miR-194 inhibits talin2 protein expression and binds to the talin2 3′-UTR (Fig. 3). In addition to regulating talin2, we have also observed that miR-194 expression reduces profilin2, another cytoskeletal protein (data not shown). However, miR-194 did not bind directly to the profilin2 3′-UTR in a luciferase reporter assay. miR-194 has been shown by others to target migration-related proteins including N-cadherin, Rac1, heparin-binding epidermal growth factor–like growth factor, type 1 insulin-like growth factor receptor, protein tyrosine phosphatase-non receptor type 12, integrin-alpha 9, suppressor of cytokine signaling 2, and BMI1 polycomb ring finger oncogene [54], [56], [58]. Inhibition of these other cytoskeletal and migration-related proteins may contribute to miR-194-induced inhibition of cell migration/invasion as well.

Talin1 and talin2 are actin-binding cytoskeletal proteins that play key roles in cell signaling, adhesion, and migration [48], [49], [59], [60]. Both talin1proteins are closely coupled to vinculin, another cytoskeletal protein found in focal adhesion complexes [49]. Both talin1 and talin2 (74% identity) can bind to integrins and to F actin via FERM (four-point-one, ezrin, radixin, moesin) domains and are important regulators of integrin activation [59], [60], [61], [62]. Genetic knockout of talin1 alone does not affect fibroblast spreading due to the compensatory activity of talin2, but does impair cytoskeletal organization [63]. Double deletion of talin1 and talin2 abolishes extracellular matrix–cytoskeletal linkage through integrins and blocks assembly of focal adhesions [64]. Talin1 knockdown in the absence of talin2 compensation has been shown to prevent spreading of endothelial cells and block angiogenesis leading to embryonic death [65]. Forced expression of talin1 enhances migration and invasion of prostate cancer cells [66]. Talin1 overexpression is a poor prognostic factor in prostate [66], liver [67] and oral [68] cancers. The role of talin2 in cancer cells including in breast cancer has not yet been reported. This study indicates that suppression of talin2 can inhibit breast cancer cell migration.

Trastuzumab treatment seems to exert its anti-tumor effect through multiple mechanisms including inducing miRNAs. After completing this study, one report has been just published and shown that trastuzumab treatment can induce miR-26a and miR-30b, leading to inhibition of cylcin E2 expression and G1 arrest of the cell cycle [69]. Our study has identified another miRNA (miR-194) and new target (talin 2, motility) in HER2-overexpressing breast cancer cells.

In conclusion, our study describes a novel mechanism of trastuzumab action in breast cancer cells. Treatment with trastuzumab can activate miR-194 expression, downregulate cytoskeletal protein talin2 expression and inhibit cell migration/invasion in HER2-overexpressing breast cancer cells.

## Supporting Information

Figure S1 **Heatmap of the differentially expressed miRNAs in response to trastuzumab treatment in HER2-overexpressing SKBr3 cells (A) and BT474 cells (B).** To visualize the clusters of expressed miRNAs after trastuzumab treatment, hierarchical clustering was performed based on the differentially expressed miRNAs. Names of samples (top) and miRNAs (side) is shown by using One Minus Correlation as a distance measure after genes centering and scaling, and average linkage method for defining the distance between the clusters. Sample BRC20, SKBr3 cells treated control hIgG for 20 hrs; BRHCT20, SKBr3 cells treated trastuzumab for 20 hrs; BTC48, BT474 cells treated with control hIgG for 48 hrs; BTHCT48, BT474 cells treated with trastuzumab for 48 hrs. A color scale of expression is shown between panel A and B.
(TIF)

Figure S2 **Effects of trastuzumab on miR-194 expression in the MDA-MB-231 cells with low-HER2 level and the KPL4 cells with high-HER2 level.** Two trastuzumab-insensitive breast cancer cell lines MDA-MB-231 (**A**) and KPL4 (**B**) were treated with trastuzumab (Tras) at different concentrations (0, 5, 10 µg/ml) for 48 hrs. Total RNA was prepared and analyzed by QRT-PCR in triplicate for miR-194 levels.
(TIF)

## References

[1] Hudziak RM, Lewis GD, Winget M, Fendly BM, Shepard HM (1989). p185HER2 monoclonal antibody has antiproliferative effects in vitro and sensitizes human breast tumor cells to tumor necrosis factor.. Mol Cell Biol.

[2] Cobleigh MA, Vogel CL, Tripathy D, Robert NJ, Scholl S (1999). Multinational study of the efficacy and safety of humanized anti-HER2 monoclonal antibody in women who have HER2-overexpressing metastatic breast cancer that has progressed after chemotherapy for metastatic disease.. J Clin Oncol.

[3] Vogel CL, Cobleigh MA, Tripathy D, Gutheil JC, Harris LN (2002). Efficacy and safety of trastuzumab as a single agent in first-line treatment of HER2-overexpressing metastatic breast cancer.. J Clin Oncol.

[4] Burstein HJ, Kuter I, Campos SM, Gelman RS, Tribou L (2001). Clinical activity of trastuzumab and vinorelbine in women with HER2-overexpressing metastatic breast cancer.. J Clin Oncol.

[5] Seidman AD, Fornier MN, Esteva FJ, Tan L, Kaptain S (2001). Weekly trastuzumab and paclitaxel therapy for metastatic breast cancer with analysis of efficacy by HER2 immunophenotype and gene amplification.. J Clin Oncol.

[6] Slamon DJ, Leyland-Jones B, Shak S, Fuchs H, Paton V (2001). Use of chemotherapy plus a monoclonal antibody against HER2 for metastatic breast cancer that overexpresses HER2.. N Engl J Med.

[7] Esteva FJ, Valero V, Booser D, Guerra LT, Murray JL (2002). Phase II study of weekly docetaxel and trastuzumab for patients with HER-2-overexpressing metastatic breast cancer.. J Clin Oncol.

[8] Hortobagyi GN, Perez EA (2001). Integration of trastuzumab into adjuvant systemic therapy of breast cancer: ongoing and planned clinical trials.. Semin Oncol.

[9] Cardoso F, Piccart MJ, Durbecq V, Di Leo A (2002). Resistance to trastuzumab: a necessary evil or a temporary challenge?. Clin Breast Cancer 3: 247–257; discussion 258–249.

[10] Nahta R, Esteva FJ (2006). HER2 therapy: molecular mechanisms of trastuzumab resistance.. Breast Cancer Res.

[11] Lane HA, Motoyama AB, Beuvink I, Hynes NE (2001). Modulation of p27/Cdk2 complex formation through 4D5-mediated inhibition of HER2 receptor signaling.. Ann Oncol.

[12] Le XF, Bedrosian I, Mao W, Murray M, Lu Z (2006). Anti-HER2 antibody trastuzumab inhibits CDK2-mediated NPAT and histone H4 expression via the PI3K pathway.. Cell Cycle.

[13] Le XF, Claret FX, Lammayot A, Tian L, Deshpande D (2003). The role of cyclin-dependent kinase inhibitor p27Kip1 in anti-HER2 antibody-induced G1 cell cycle arrest and tumor growth inhibition.. J Biol Chem.

[14] Le XF, Lammayot A, Gold D, Lu Y, Mao W (2005). Genes affecting the cell cycle, growth, maintenance, and drug sensitivity are preferentially regulated by anti-HER2 antibody through phosphatidylinositol 3-kinase-AKT signaling.. J Biol Chem.

[15] Le XF, McWatters A, Wiener J, Wu JY, Mills GB (2000). Anti-HER2 antibody and heregulin suppress growth of HER2-overexpressing human breast cancer cells through different mechanisms.. Clin Cancer Res.

[16] Le XF, Pruefer F, Bast RC (2005). HER2-targeting antibodies modulate the cyclin-dependent kinase inhibitor p27Kip1 via multiple signaling pathways.. Cell Cycle.

[17] Le XF, Vadlamudi R, McWatters A, Bae DS, Mills GB (2000). Differential signaling by an anti-p185(HER2) antibody and heregulin.. Cancer Res.

[18] Junttila TT, Akita RW, Parsons K, Fields C, Lewis Phillips GD (2009). Ligand-independent HER2/HER3/PI3K complex is disrupted by trastuzumab and is effectively inhibited by the PI3K inhibitor GDC-0941.. Cancer Cell.

[19] Yakes FM, Chinratanalab W, Ritter CA, King W, Seelig S (2002). Herceptin-induced inhibition of phosphatidylinositol-3 kinase and Akt Is required for antibody-mediated effects on p27, cyclin D1, and antitumor action.. Cancer Res.

[20] Nagata Y, Lan KH, Zhou X, Tan M, Esteva FJ (2004). PTEN activation contributes to tumor inhibition by trastuzumab, and loss of PTEN predicts trastuzumab resistance in patients.. Cancer Cell.

[21] Pegram MD, Slamon DJ (1999). Combination therapy with trastuzumab (Herceptin) and cisplatin for chemoresistant metastatic breast cancer: evidence for receptor-enhanced chemosensitivity.. Semin Oncol.

[22] Wen XF, Yang G, Mao W, Thornton A, Liu J (2006). HER2 signaling modulates the equilibrium between pro- and antiangiogenic factors via distinct pathways: implications for HER2-targeted antibody therapy.. Oncogene.

[23] Guler M, Yilmaz T, Ozercan I, Elkiran T (2009). The inhibitory effects of trastuzumab on corneal neovascularization.. Am J Ophthalmol 147: 703–708 e702.

[24] Le XF, Mao W, Lu C, Thornton A, Heymach JV (2008). Specific blockade of VEGF and HER2 pathways results in greater growth inhibition of breast cancer xenografts that overexpress HER2.. Cell Cycle.

[25] Klos KS, Zhou X, Lee S, Zhang L, Yang W (2003). Combined trastuzumab and paclitaxel treatment better inhibits ErbB-2-mediated angiogenesis in breast carcinoma through a more effective inhibition of Akt than either treatment alone.. Cancer.

[26] Koukourakis MI, Simopoulos C, Polychronidis A, Perente S, Botaitis S (2003). The effect of trastuzumab/docatexel combination on breast cancer angiogenesis: dichotomus effect predictable by the HIFI alpha/VEGF pre-treatment status?. Anticancer Res.

[27] Izumi Y, Xu L, di Tomaso E, Fukumura D, Jain RK (2002). Tumour biology: herceptin acts as an anti-angiogenic cocktail.. Nature.

[28] Baselga J, Albanell J (2001). Mechanism of action of anti-HER2 monoclonal antibodies.. Ann Oncol.

[29] Molina MA, Codony-Servat J, Albanell J, Rojo F, Arribas J (2001). Trastuzumab (herceptin), a humanized anti-Her2 receptor monoclonal antibody, inhibits basal and activated Her2 ectodomain cleavage in breast cancer cells.. Cancer Res.

[30] Arnould L, Gelly M, Penault-Llorca F, Benoit L, Bonnetain F (2006). Trastuzumab-based treatment of HER2-positive breast cancer: an antibody-dependent cellular cytotoxicity mechanism?. Br J Cancer.

[31] Beano A, Signorino E, Evangelista A, Brusa D, Mistrangelo M (2008). Correlation between NK function and response to trastuzumab in metastatic breast cancer patients.. J Transl Med.

[32] Kono K, Takahashi A, Ichihara F, Sugai H, Fujii H (2002). Impaired antibody-dependent cellular cytotoxicity mediated by herceptin in patients with gastric cancer.. Cancer Res.

[33] Lee S, Yang W, Lan KH, Sellappan S, Klos K (2002). Enhanced sensitization to taxol-induced apoptosis by herceptin pretreatment in ErbB2-overexpressing breast cancer cells.. Cancer Res.

[34] Henson ES, Hu X, Gibson SB (2006). Herceptin sensitizes ErbB2-overexpressing cells to apoptosis by reducing antiapoptotic Mcl-1 expression.. Clin Cancer Res.

[35] Mohsin SK, Weiss HL, Gutierrez MC, Chamness GC, Schiff R (2005). Neoadjuvant trastuzumab induces apoptosis in primary breast cancers.. J Clin Oncol.

[36] Ambros V (2001). microRNAs: tiny regulators with great potential.. Cell.

[37] He L, Hannon GJ (2004). MicroRNAs: small RNAs with a big role in gene regulation.. Nat Rev Genet.

[38] Spizzo R, Nicoloso MS, Croce CM, Calin GA (2009). SnapShot: MicroRNAs in Cancer.. Cell 137: 586–586 e581.

[39] Nicoloso MS, Spizzo R, Shimizu M, Rossi S, Calin GA (2009). MicroRNAs–the micro steering wheel of tumour metastases.. Nat Rev Cancer.

[40] Le XF, Merchant O, Bast RC, Calin GA (2010). The Roles of MicroRNAs in the Cancer Invasion-Metastasis Cascade.. Cancer Microenviron.

[41] Hummel R, Hussey DJ, Haier J (2010). MicroRNAs: predictors and modifiers of chemo- and radiotherapy in different tumour types.. Eur J Cancer.

[42] Rukov JL, Shomron N (2011). MicroRNA pharmacogenomics: post-transcriptional regulation of drug response.. Trends Mol Med.

[43] Allen KE, Weiss GJ (2010). Resistance may not be futile: microRNA biomarkers for chemoresistance and potential therapeutics.. Mol Cancer Ther.

[44] Calin GA, Dumitru CD, Shimizu M, Bichi R, Zupo S (2002). Frequent deletions and down-regulation of micro- RNA genes miR15 and miR16 at 13q14 in chronic lymphocytic leukemia.. Proc Natl Acad Sci U S A.

[45] Le XF, Arachchige-Don AS, Mao W, Horne MC, Bast RC (2007). Roles of human epidermal growth factor receptor 2, c-jun NH2-terminal kinase, phosphoinositide 3-kinase, and p70 S6 kinase pathways in regulation of cyclin G2 expression in human breast cancer cells.. Mol Cancer Ther.

[46] Le XF, Mao W, Lu Z, Carter BZ, Bast RC (2010). Dasatinib induces autophagic cell death in human ovarian cancer.. Cancer.

[47] Fabbri M, Garzon R, Cimmino A, Liu Z, Zanesi N (2007). MicroRNA-29 family reverts aberrant methylation in lung cancer by targeting DNA methyltransferases 3A and 3B.. Proc Natl Acad Sci U S A.

[48] Burridge K, Connell L (1983). Talin: a cytoskeletal component concentrated in adhesion plaques and other sites of actin-membrane interaction.. Cell Motil.

[49] Burridge K, Mangeat P (1984). An interaction between vinculin and talin.. Nature.

[50] Lu J, Getz G, Miska EA, Alvarez-Saavedra E, Lamb J (2005). MicroRNA expression profiles classify human cancers.. Nature.

[51] Barad O, Meiri E, Avniel A, Aharonov R, Barzilai A (2004). MicroRNA expression detected by oligonucleotide microarrays: system establishment and expression profiling in human tissues.. Genome Res.

[52] Hino K, Fukao T, Watanabe M (2007). Regulatory interaction of HNF1-alpha to microRNA-194 gene during intestinal epithelial cell differentiation.. Nucleic Acids Symp Ser (Oxf).

[53] Hino K, Tsuchiya K, Fukao T, Kiga K, Okamoto R (2008). Inducible expression of microRNA-194 is regulated by HNF-1alpha during intestinal epithelial cell differentiation.. Rna.

[54] Meng Z, Fu X, Chen X, Zeng S, Tian Y (2010). miR-194 is a marker of hepatic epithelial cells and suppresses metastasis of liver cancer cells in mice.. Hepatology.

[55] Braun CJ, Zhang X, Savelyeva I, Wolff S, Moll UM (2008). p53-Responsive micrornas 192 and 215 are capable of inducing cell cycle arrest.. Cancer Res.

[56] Song Y, Zhao F, Wang Z, Liu Z, Chiang Y (2011). Inverse Association between miR-194 Expression and Tumor Invasion in Gastric Cancer.. Ann Surg Oncol.

[57] Georges SA, Biery MC, Kim SY, Schelter JM, Guo J (2008). Coordinated regulation of cell cycle transcripts by p53-Inducible microRNAs, miR-192 and miR-215.. Cancer Res.

[58] Dong P, Kaneuchi M, Watari H, Hamada J, Sudo S (2011). MicroRNA-194 inhibits epithelial to mesenchymal transition of endometrial cancer cells by targeting oncogene BMI-1.. Mol Cancer.

[59] Franco-Cea A, Ellis SJ, Fairchild MJ, Yuan L, Cheung TY (2010). Distinct developmental roles for direct and indirect talin-mediated linkage to actin.. Dev Biol.

[60] Moser M, Legate KR, Zent R, Fassler R (2009). The tail of integrins, talin, and kindlins.. Science.

[61] Monkley SJ, Pritchard CA, Critchley DR (2001). Analysis of the mammalian talin2 gene TLN2.. Biochem Biophys Res Commun.

[62] Di Paolo G, Pellegrini L, Letinic K, Cestra G, Zoncu R (2002). Recruitment and regulation of phosphatidylinositol phosphate kinase type 1 gamma by the FERM domain of talin.. Nature.

[63] Priddle H, Hemmings L, Monkley S, Woods A, Patel B (1998). Disruption of the talin gene compromises focal adhesion assembly in undifferentiated but not differentiated embryonic stem cells.. J Cell Biol.

[64] Zhang X, Jiang G, Cai Y, Monkley SJ, Critchley DR (2008). Talin depletion reveals independence of initial cell spreading from integrin activation and traction.. Nat Cell Biol.

[65] Monkley SJ, Kostourou V, Spence L, Petrich B, Coleman S (2010). Endothelial cell talin1 is essential for embryonic angiogenesis.. Dev Biol.

[66] Sakamoto S, McCann RO, Dhir R, Kyprianou N (2010). Talin1 promotes tumor invasion and metastasis via focal adhesion signaling and anoikis resistance.. Cancer Res.

[67] Kanamori H, Kawakami T, Effendi K, Yamazaki K, Mori T (2011). Identification by differential tissue proteome analysis of talin-1 as a novel molecular marker of progression of hepatocellular carcinoma.. Oncology.

[68] Sansing HA, Sarkeshik A, Yates JR, Patel V, Gutkind JS (2011). Integrin alphabeta1, alphavbeta, alpha6beta effectors p130Cas, Src and talin regulate carcinoma invasion and chemoresistance.. Biochem Biophys Res Commun.

[69] Ichikawa T, Sato F, Terasawa K, Tsuchiya S, Toi M (2012). Trastuzumab Produces Therapeutic Actions by Upregulating miR-26a and miR-30b in Breast Cancer Cells.. PLoS One.

