# Supplementary figures and images for: Modulation of MicroRNA-194 and Cell Migration by HER2-Targeting Trastuzumab in Breast Cancer
Source: PLoS One. 2012 Jul 19;7(7):e41170. doi: 10.1371/journal.pone.0041170 (PMC3400637; doi:10.1371/journal.pone.0041170)

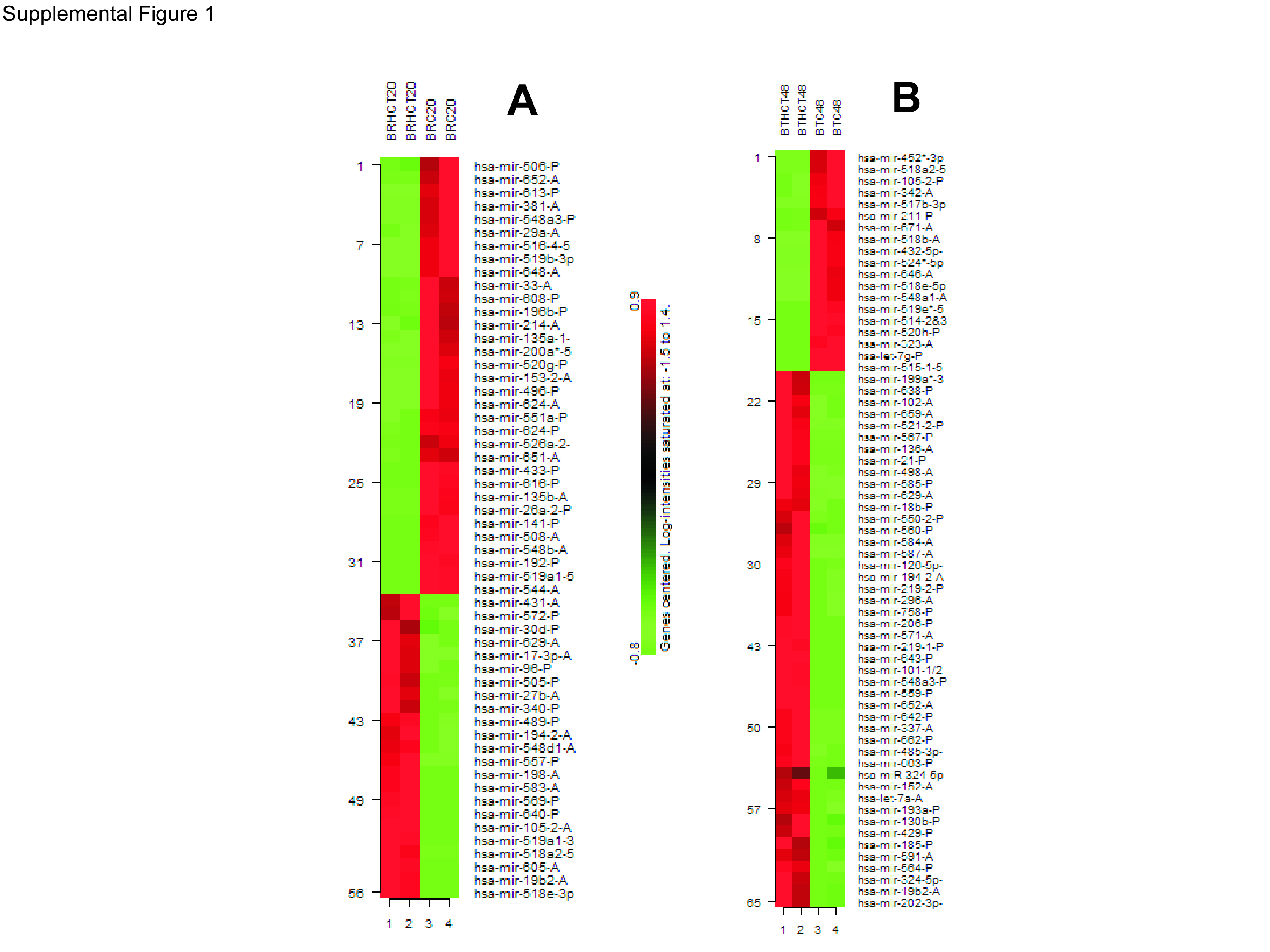

Supplement: Figure S1 — Heatmap of the differentially expressed miRNAs in response to trastuzumab treatment in HER2-overexpressing SKBr3 cells (A) and BT474 cells (B). To visualize the clusters of expressed miRNAs after trastuzumab treatment, hierarchical clustering was performed based on the differentially expressed miRNAs. Names of samples (top) and miRNAs (side) is shown by using One Minus Correlation as a distance measure after genes centering and scaling, and average linkage method for defining the distance between the clusters. Sample BRC20, SKBr3 cells treated control hIgG for 20 hrs; BRHCT20, SKBr3 cells treated trastuzumab for 20 hrs; BTC48, BT474 cells treated with control hIgG for 48 hrs; BTHCT48, BT474 cells treated with trastuzumab for 48 hrs. A color scale of expression is shown between panel A and B. (TIF) [file pone.0041170.s001.tif]

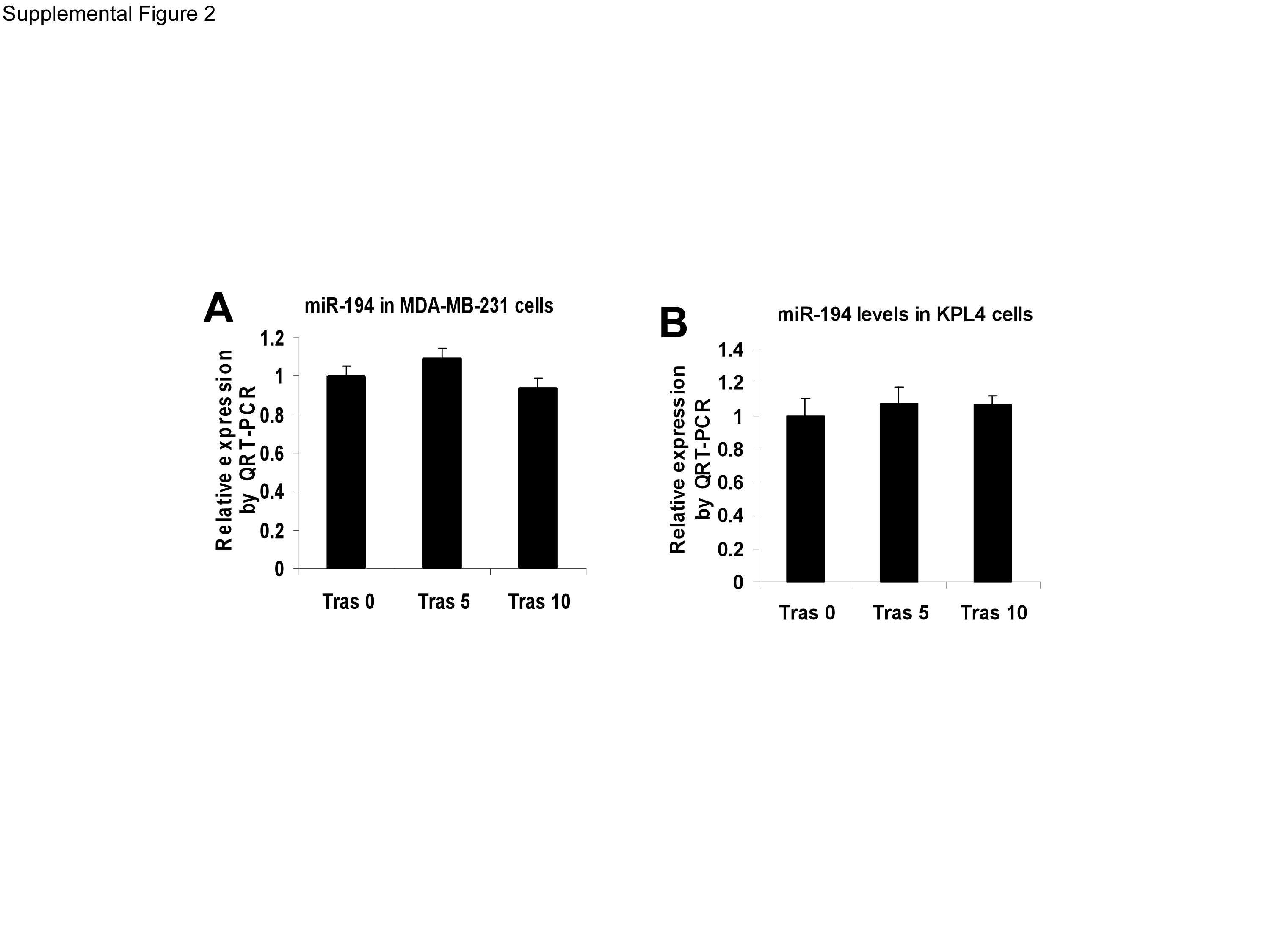

Supplement: Figure S2 — Effects of trastuzumab on miR-194 expression in the MDA-MB-231 cells with low-HER2 level and the KPL4 cells with high-HER2 level. Two trastuzumab-insensitive breast cancer cell lines MDA-MB-231 (A) and KPL4 (B) were treated with trastuzumab (Tras) at different concentrations (0, 5, 10 µg/ml) for 48 hrs. Total RNA was prepared and analyzed by QRT-PCR in triplicate for miR-194 levels. (TIF) [file pone.0041170.s002.tif]
